# Factors Associated with Readmission of Patients at a University Hospital Psychiatric Ward in Iran

**DOI:** 10.1155/2013/685625

**Published:** 2013-03-17

**Authors:** Majid Barekatain, Mohammad Reza Maracy, Razeyeh Hassannejad, Reihane Hosseini

**Affiliations:** ^1^Behavioral Sciences Research Center, Isfahan University of Medical Sciences, Hezar Jerib Street, Isfahan 8169658514, Iran; ^2^Depatment of Epidemiology and Biostatistics, Behavioral Sciences Research Center, Isfahan University of Medical Sciences, Hezar Jerib Street, Isfahan 8169658514, Iran; ^3^Depatment of Epidemiology and Biostatistics, Isfahan University of Medical Sciences, Hezar Jerib Street, Isfahan 8169658514, Iran

## Abstract

*Objectives*. Readmission has a major role in the reduction of the quality of life and the increase in the years of lost life. The main objectives of this study were to answer to the following research questions. (a) What was the readmission rate? (b) What were the social, demographic, and clinical characteristics of patients admitted to the Psychiatric Emergency Service at Nour University Hospital, affiliated to Isfahan University of Medical Sciences, Isfahan, Iran? (c) What were the effective factors on readmission? *Method*. This cross-sectional study was conducted on a total number of 3935 patients who were admitted to Isfahan University Hospital Psychiatric Ward in Isfahan, Iran, from 2004 to 2010. Gender, age, marital status, education, self-report history of previous admission, type of psychiatric disorder, substance misuse, suicide, and the length of the current psychiatric disorder were collected from the registered medical files of patients. The data were analysed using the negative binomial regression model. *Results*. We found that factors such as psychiatric anxiety disorder, bipolar I, bipolar II, psychotic disorder, depression, and self report history of previous admission were statistically significant in the number of readmissions using the negative binomial model. *Conclusion*. Readmission to the psychiatric ward is mainly predictable by the type of diagnosis and psychosocial supports.

## 1. Introduction

During the last three decades, the deinstitutionalization of mental health services led to faster transitions from psychiatric hospitals to the community. However, a significant number of patients have had serious problems after being discharged from hospitals which resulted in an increase in the number of psychiatric emergency referrals and readmissions [1]. The greater the number of readmissions, the greater the pressure on hospitals psychiatric wards and their limited resources. 

Readmission of inpatients has been one of the most important problems in the field of psychiatry for the last decades [2, 3]. The problem has a major role in reducing the quality of life and increasing the years of lost life [4]. About one-third of patients admitted to psychiatric services will probably be readmitted within a year [5]. In previous studies, the rate of readmitted patients has proven to be 22% in America in psychiatric patients aged over 65 years [4], 19% of children admitted at the emergency ward in Baltimore were aged 4–18 [6], 14% of patients admitted at the psychological ward of the army in Washington [7], 33.7% of patients diagnosed with schizophrenia in Turkey [8], and 43% of teenage patients admitted for psychological reasons in Northern Carolina [3]. The readmission period has been defined controversially in different studies, for example, readmission in a period of 90 days [9], readmission in a period of 3 years [10], admission of three or more times in a period of 30 months [11], readmission in a period of 6 months [12], and admittance of 3 or more times in a period of 2.5 years [13]. This, in turn, resulted in different rates of readmission in different studies as 14% [7], 16% [12], 20–30% [10], and 45–53% [13].

Studying the characteristics related to readmission may help to improve management programs. The factors such as demographics, social, and clinical factors have been studied for more than three decades [14]. In addition, poor adherence to treatment [8, 15–17], nonvoluntary first admission [14, 17], substance, alcohol, and drug abuse [4, 7, 14, 16] have been mentioned as related factors to the readmission of patients to psychiatric wards. Some studies have illustrated that diagnoses are related to readmission. For example, the diagnosis of schizophrenia has been reported as a major factor on readmission [4, 15, 17–20].

Length of stay at hospital has also been notified as a highly related factor [15, 17, 19, 21]. The shorter the length of the stay at a hospital, due to the lack of empty beds, the more readmission of psychiatric patients. This could decrease the quality of care and definitely increase the costs [10]. In some studies, the history of admission in elderly people has been pointed as a related factor [6, 7, 15, 18, 19]. The diagnosis of bipolar disorder [4, 14], psychotic disorders [7, 21], and mood disorders [3, 7] is also mentioned as an effective factors on readmission.

From the demographic characteristics marital status, unemployment [18, 19], retirement [19], and gender [8, 18] are the most relevant factors to readmission. In some studies, being diagnosed with more psychiatric comorbidities [6] was also a factor associated with readmission. 

Suicide [6], history of psychological problems in childhood, and first episode of the psychiatric disorder under age of 18 years have been revealed as related factors to readmission [7].

Regarding the mentioned findings, we need a better understanding of the factors related to readmission to help better management and early intervention. Understanding these factors will decrease the number of readmissions and hospital costs. It seems that those inpatients who are seeking rehospitalisation tend to repeat some of these treatment-seeking behaviors and may assist nurses in planning care [2, 4, 6, 14, 15].

In more detail, we had the following research questions. (a) What was the readmission rate? (b) What were the social, demographic, and clinical characteristics of patients admitted to Psychiatric Emergency Service at Nour University Hospital, affiliated to Isfahan University of Medical Sciences, Isfahan, Iran? (c) What were the effective factors on readmission? We try to answer these three questions in Section 3.

## 2. Material and Method

In this cross-sectional study, data were collected from the medical files of 3935 patients with psychiatric disorders who were admitted to the Nour Hospital Psychiatric Ward affiliated to Isfahan University of Medical Sciences, Isfahan, Iran, from September 2004 to September 2010. The patients were diagnosed to have at least one major psychiatric disorder based on Diagnostic and Statistical Manual of Mental Disorders, the fourth edition (DSM-IV) criteria. The major diagnoses were bipolar one disorder (BID), bipolar two disorder (BIID), bipolar disorder not otherwise specified (BD-NOS), psychotic disorders (not mood related), anxiety disorders (all types were considered as one category diagnosis), major depressive disorder (MDD), substance induced mental disorders, and cognitive disorders. All data were gathered through a semistructured interview, which was done by a board of certified attending psychiatrists and psychiatric residents. Readmitted patients were taken as the independent variable which was numeric, but for descriptive goals the number of admissions were categorised into three groups of one admission, two admissions, and three or more admissions based on the above-mentioned major diagnoses. It has been assumed that there is no possibility for the patient to change diagnosis between two admissions. Demographic characteristics such as sex, age, marital status (single, married or widowed, and divorced), and education (illiterate, primary school, high school, and higher) were also analysed. Variables such as length of the current episode before hospitalization, current substance misuse, smoking, alcohol consumption, history of admission, and history of attempt to suicide, based on the patients' self reports, were also applied. The presence or absence of each of the diagnoses, smoking, substance misuse, alcohol consumption, history of admission, and history of suicide was binary. Patients might simultaneously be diagnosed with more than one psychiatric disorder. Because the response variable (number of readmissions) was numeric without normal distribution and small spread within data, the negative binomial regression model was used to analyze the data set to determine the factors related to readmission. A *P* value of less than 0.05 was considered as significant.

## 3. Results

Out of the 3935 inpatients, 2158 (54.8%) were male. The age mean and median were 33.1 and 30 years, respectively, with a range of 14–86 years. The 95% confidence interval (CI) of the age of the patients was 32.7–33.5. The mean (95% CI) of age in male and female patients were 33.8 (33.2–34.4) and 32.5 (31.9–33.1), respectively. There was a statistically significant difference between males and females' mean of age (*P* value = 0.002) (results not shown). The readmission rate of inpatients varied between 1 and 10 times in the number of previous admissions. Of the total number of inpatients, 3235 (82.3%) had only one admission, 501 (12.7%) were admitted two times, 126 (3.2%) had three times, and 75 (1.9%) were admitted four or more times.  Table 1 is referred to the first research question. The results showed that there is no significant difference between male and female regarding readmission rate (*P* value > 0.05) (results not shown). 

From the study population, 1541 (39.2%) patients were diagnosed with BID, 1119 (28.4%) with nonmood psychotic disorder, 626 (15.9%) with MDD, 471 (12%) with anxiety disorders, 279 (7.1%) with BD-NOS, 274 (7%) with BIID, 193 (4.9%) with substance induced mental disorders, and 47 (1.2%) with cognitive disorder. The frequency (%) distribution of comorbidities revealed that 3295 (83.7%) patients were only diagnosed with one disorder, 574 (14.6%) diagnosed with simultaneous two disorders, 58 (1.5%) with simultaneously three, and 8 (0.2%) with simultaneously four disorders. The number of comorbidities (other psychotic disorder) in male patients were more than that of females and the difference was statistically significant (*P* value = 0.002). Of patients with only one diagnosis, 573 (17.2%) were readmitted during the period of study. Of patients with 2 simultaneous diagnoses, 118 (21%) were readmitted through the study period. And 8 (23.5%) of patients with 3 comorbidities were readmitted during the study time. More information is presented in Table 2 which is referred to the second research question.

Negative binomial regression models were used to determine the variables related to the number of times of readmission which referred to the last research question. The results showed that being divorced had a statistically significant correlation with a higher readmission count. The mean of the number of times of admissions for divorced patients was 1.47 times and 1.56 times more than the mean for single patients and married patients, respectively. The education status of the patients had no statistically significant correlation with the readmission rate. 

Regarding diagnoses, BID, BIID, psychotic disorders (not mood related), MDD, and anxiety disorders showed to be significantly related to the number of times of readmission. However, BID-NOS, cognitive disorders or misusing drug was not significantly related to the number of times of readmission. The mean of the number of times of readmission in patients who misused opium was about 1.3 times more than in patients who did not (*P* = 0.045). 

The results showed a significant relationship between self report history of a previous admission and the current admission of patients. The mean of the number of times of a current admission in patients with positive history of admission was about 2.2 times more than patients without self report of previous history of admission (*P* < 0.001). The results indicate that there was no statistically significant relationship between the number of times of readmission and the length of the current episode before hospitalization. More information about the results of fitting the negative binomial regression model is illustrated in Table 3. 

## 4. Discussion

The reported factors related to the readmission of patients with psychiatric disorders were various due to objective limitations, type of studies [2, 17], or type of models used for analyzing the data. This study showed that the type of diagnosis was significantly associated with the number of readmissions and all types of the above-mentioned psychiatric disorders, except bipolar NOS and cognitive disorders. Some studies indicated a relationship between schizophrenia and the number of readmissions [4, 15, 18–20]. In our study, schizophrenia was also mentioned as a subcategory of psychotic disorders (not mood related). Other studies revealed that bipolar disorder increases the number readmissions [4, 14]. In the current work, BID and BIID, but not bipolar disorder NOS, were significantly related to readmission. 

The length of the current episode was not identified as a statistically significant factor for consequent rehospitalization. However, in one study, length of stay at hospital was a predictor of referral to the aftercare in patients who had been diagnosed for schizophrenia [12]. 

Some studies showed that the history of previous admission may lead to an increase in the number of readmissions [6, 7, 15, 18, 19]. In one study, patients with history of previous admission had a greater number of readmissions in comparison to patients without such history [2]. Our study revealed the same results.

In conclusion, the readmission to the psychiatric ward is mainly predictable by the type of diagnosis and psychosocial supports. The results of this study could be taken into account to help better manage the admission of patients seeking rehospitalisation. It may also lead to a better understanding of early intervention and may assist nurses in planning care. 

The first weaknesses of our study was that the Global Assessment Functioning (GAF), as one the important predictors of outcome measures, was not determined [22]. The second, was not being able to assess accurately the out-of-network hospitalization experience in the community. It means this could lead to a selection bias, and as a result it may affect the outcome of this sample. Therefore, the generalization of our findings to other groups or communities is uncertain. As a limitation, we assumed that there is no possibility for the patient to change diagnosis between two admissions.

## Figures and Tables

**Table 1:** The frequency (%) distribution of admissions number at Nour (Psychological Ward) Hospital, Isfahan, Iran, from 2004 to 2010.

Admission	1	2	3	4	5	6	7	8	9	10
Number	3235	501	126	44	16	5	4	0	3	1
Percentage	82.21	12.73	3.20	1.12	0.41	0.13	0.10	0	0.08	0.02

**Table 2:** The frequency (column%) distribution of characteristics and psychiatric disorder patients based on the number of admission at Nour (the Psychological Ward) Hospital, Isfahan, Iran from 2004 to 2010.

Characteristics		Number of patients	Number of admission			*P* value
			One time (%)	Two times (%)	≥three times (%)	
Sex	Male	2158	1774 (54.9)	275 (55)	109 (55.1)	0.998
	Female	1777	1461 (45.1)	226 (45)	90 (44.9)	
Age	<20 years	339	278 (8.6)	44 (8.8)	17 (8.5)	0.400
	20–39	2499	2045 (63.3)	330 (65.9)	124 (62.3)	
	40–59	931	769 (23.8)	111 (22.2)	51 (25.6)	
	≥60	161	138 (4.3)	16 (3.2)	7 (3.5)	
Material status	Single	1608	1319 (40.8)	204 (40.7)	85 (42.7)	0.009
	Marriage	2082	1735 (53.7)	249 (49.7)	98 (49.2)	
	Divorce	243	179 (5.5)	48 (9.6)	16 (8.1)	
Education	Illiterate	272	228 (7.2)	30 (6)	14 (7.1)	
	Primary and secondary	1071	869 (27.4)	124 (24.8)	51 (25.7)	.121
	High school	2045	1661 (52.3)	275 (55)	109 (55.1)	
	Higher	510	415 (13.1)	71 (14.2)	24 (12.1)	
Type of disorder	Bipolar I	1544	1243 (38.4)	208 (41.6)	93 (46.7)	0.037
	Psychotic	1119	904 (27.9)	154 (30.8)	61 (30.6)	0.311
	Depression	626	521 (16.1)	79 (15.8)	26 (13.1)	0.525
	Anxiety	471	381 (11.8)	58 (11.6)	32 (16.1)	0.187
	Bipolar NOS	279	240 (7.4)	31 (6.2)	8 (4)	0.139
	Bipolar II	274	228 (7.1)	33 (6.6)	13 (6.5)	0.912
	Cognitive or misusing drugs	251	210 (6.5)	30 (6)	11 (5.5)	0.823
Alcohol	Yes	229	183 (5.6)	33 (6.6)	13 (6.5)	0.631
Smoking	Yes	1124	907 (28.1)	146 (29.2)	71 (35.7)	0.067
Cannabis	Yes	147	124 (3.8)	15 (3)	8 (4)	0.649
Opium	Yes	380	303 (9.4)	54 (10.8)	23 (11.6)	0.445
Self-report history of previous admissions	Yes	617	431 (13.3)	117 (23.4)	69 (34.7)	<0.001
Self report history of suicide	Yes	442	366 (11.3)	55 (11)	21 (10.6)	0.902
Length of the current episode before hospitalization	<1 month	1935	1577 (48.9)	254 (50.8)	104 (52.3)	0.092
	1–6	1581	1295 (40.1)	207 (41.4)	79 (39.7)	
	>6	409	354 (11)	39 (7.8)	16 (8)	

**Table 3:** Results from fitting model of the negative binomial regression of psychiatric disorder patients at Nour (the Psychological Ward) Hospital, Isfahan, Iran, from 2004 to 2010.

Characteristics		Negative binomial regression		*P* value
		Exp. (*β*)	95% CI	
Marital status	Single	0.68	(0.52, 0.81)	0.005
	Marriage	0.64	(0.49, 0.83)	0.001
	Widow	1	—	—
Education	Illiterate	1	—	—
	Primary and secondary	0.85	(0.60, 1.93)	0.226
	High school	0.99	(0.72, 1.39)	0.874
	Higher	0.94	(0.64, 1.38)	0.614
Disorders*	Bipolar I	1.63	(1.15, 2.31)	0.003
	Psychotic	1.71	(1.19, 2.43)	0.001
	Depression	1.47	(1.01, 2.13)	0.026
	Anxiety	1.45	(1.14, 1.86)	0.001
	Bipolar NOS	1.14	(0.72, 1.81)	0.598
	Bipolar II	1.53	(1.00, 2.37)	0.036
	Cognitive	1.03	(0.72, 1.49)	0.785
	Opium:	1.28	(1.02, 1.68)	0.045
Self report history of previous admissions	Yes	2.25	(1.86, 2.72)	<0.001
Length of the current episode before hospitalization	<1 month	1	—	—
	1–6	0.98	(0.83, 1.16)	0.696
	>6	0.79	(0.59, 1.06)	0.080
*Akaikeinformation criteria (AIC) *		*4853 *		
*Bayesian information criteria (BIS) *		*4966 *		

CI: confidence interval.

*Reference is “no disorder.”
